# Supplementary material for: Regulatory B Lymphocytes Colonize the Respiratory Tract of Neonatal Mice and Modulate Immune Responses of Alveolar Macrophages to RSV Infection in IL-10-Dependant Manner
Source: Viruses. 2020 Jul 29;12(8):822. doi: 10.3390/v12080822 (PMC7472339; doi:10.3390/v12080822)
Supplement: Supplementary file 1 [file viruses-12-00822-s001.pdf]

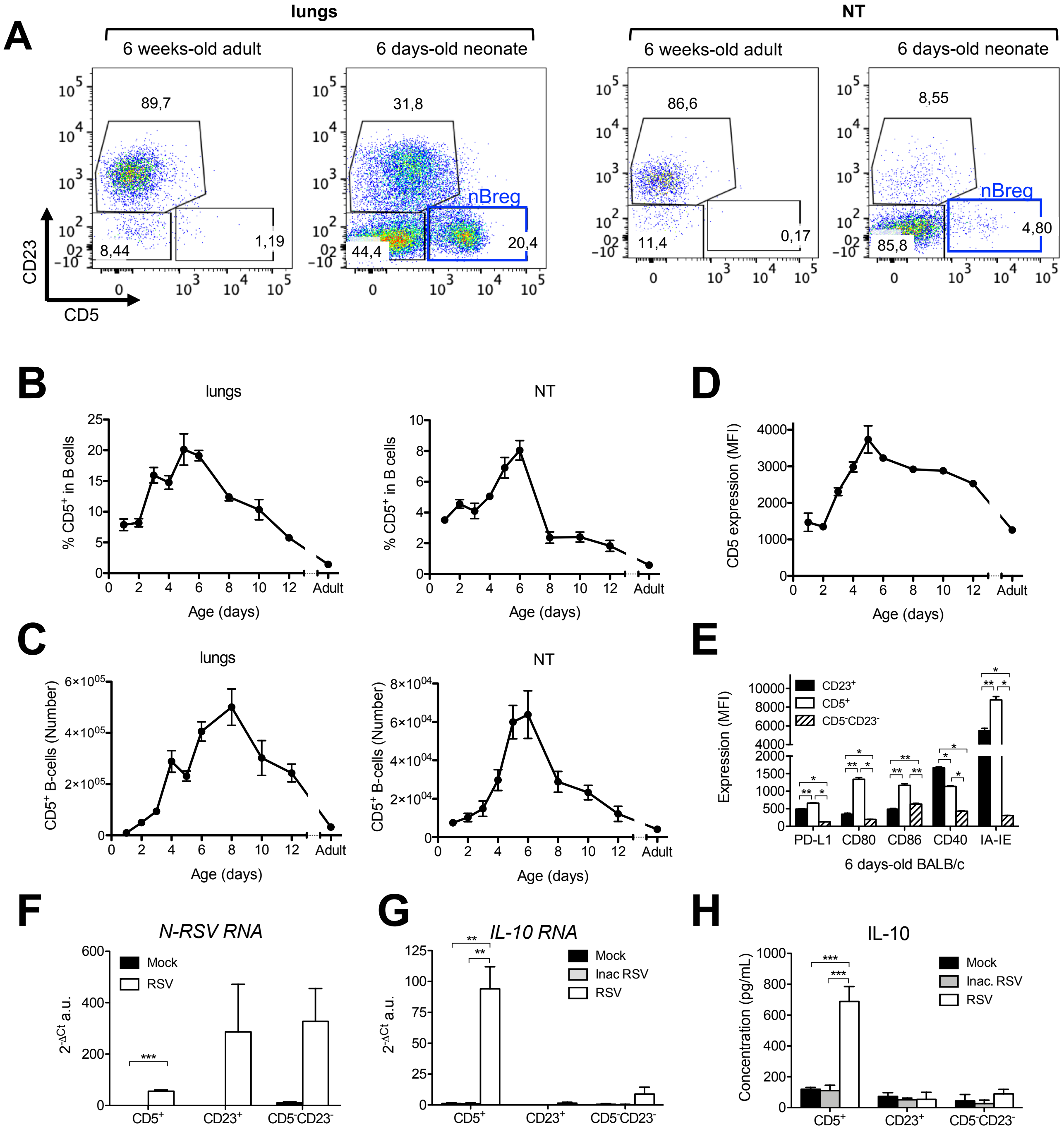

Figure 1: Neonatal regulatory B lymphocytes accumulate in the respiratory tract during the early period of life and produce IL-10 in response to RSV infection.

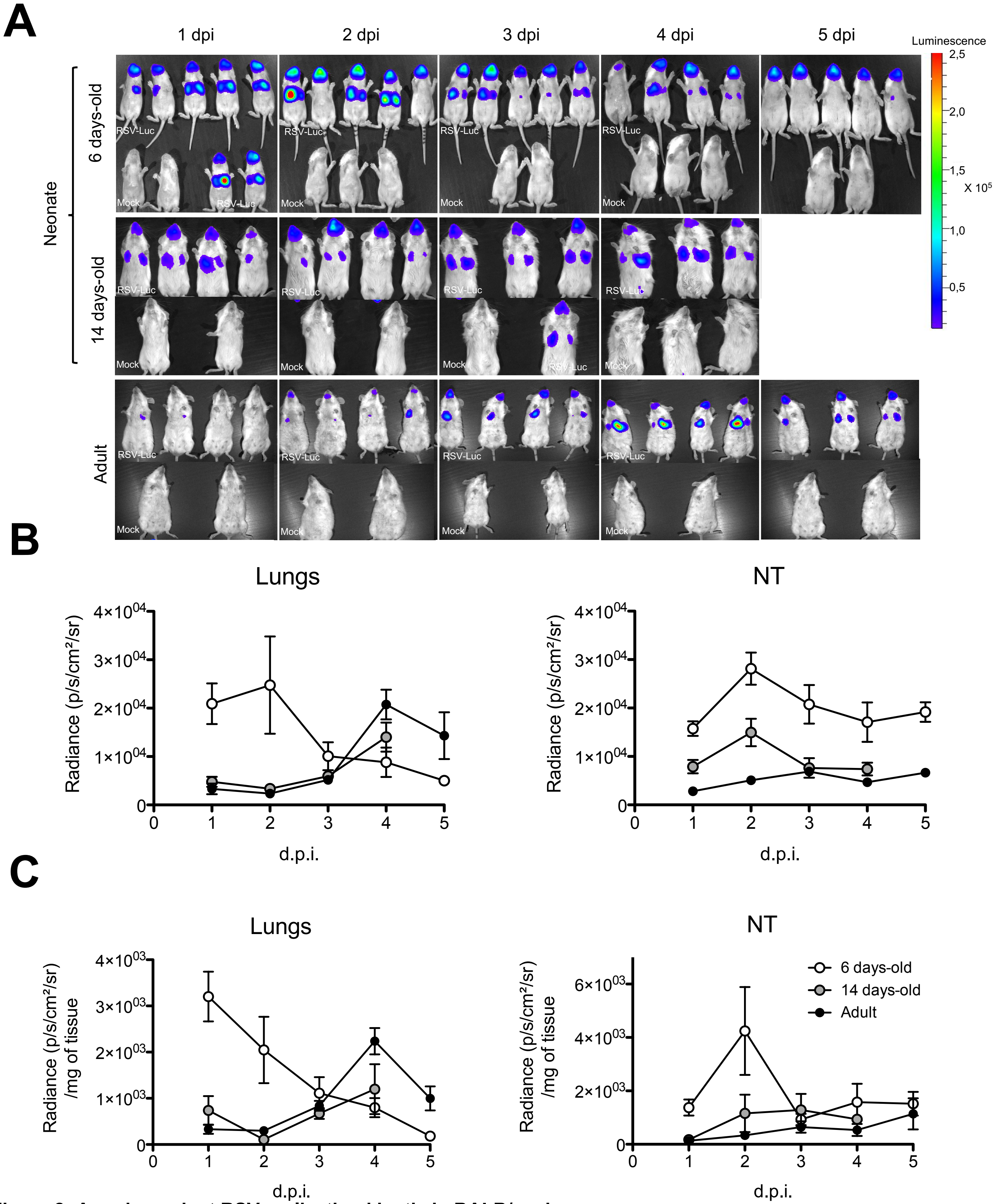

Figure 2: Age-dependent RSV replication kinetic in BALB/c mice.

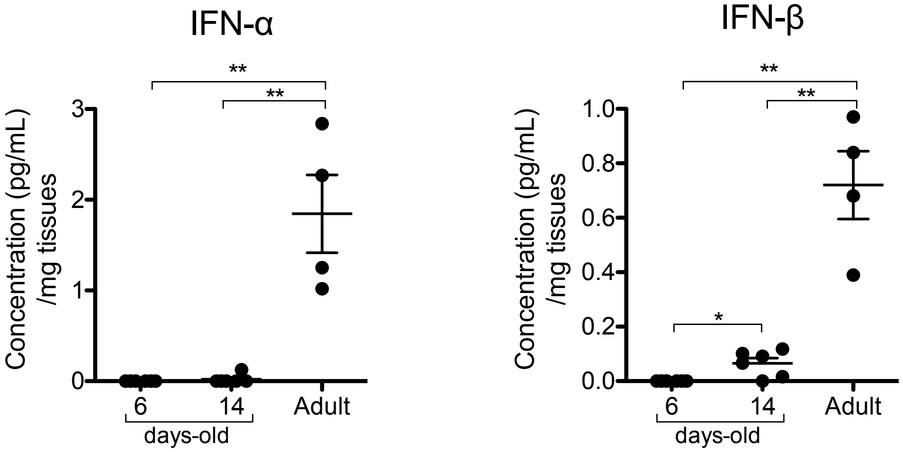

Figure 3 : Type-I interferon antiviral response is reduced in neonates.

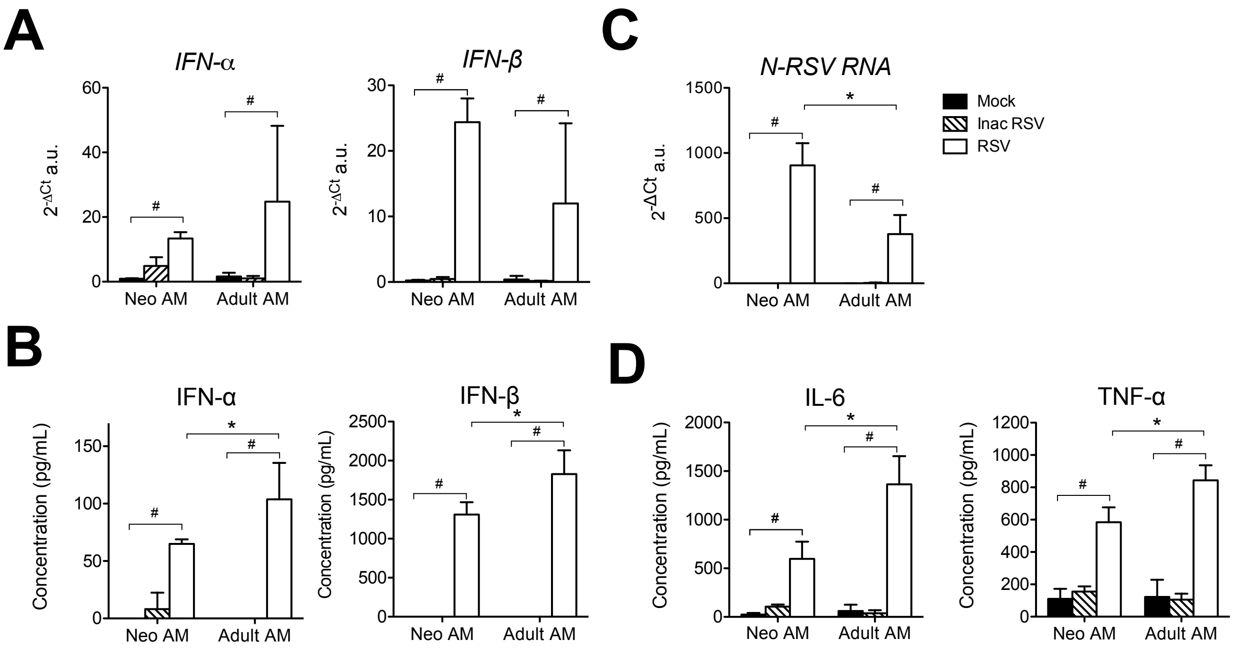

Figure 4: Neonatal primary AMs produce IFN-I and inflammatory cytokines in response to *ex vivo* RSV infection.

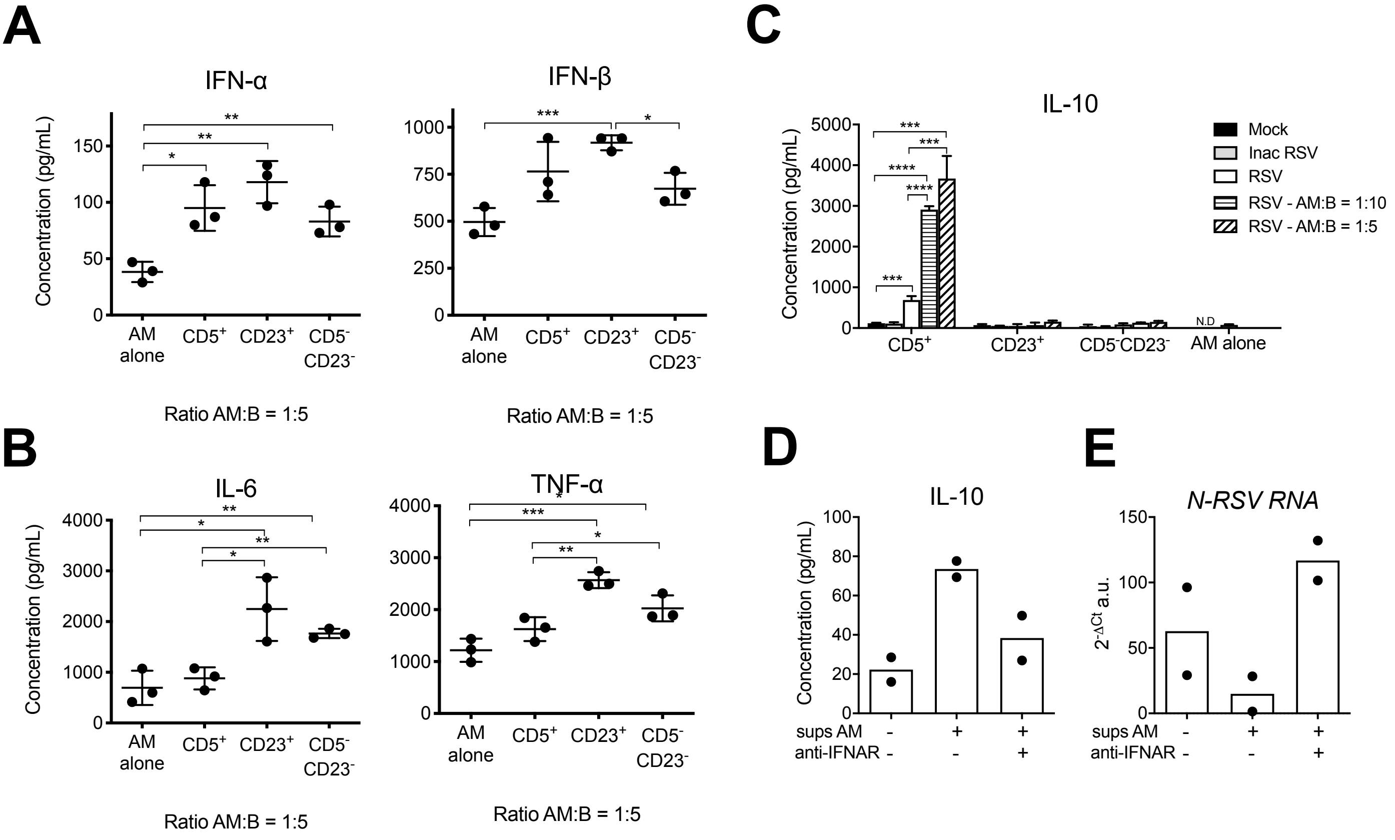

Figure 5: The IFN-I response of neonatal AMs to RSV enhanced IL-10 production by $CD5^+$ B-cells.

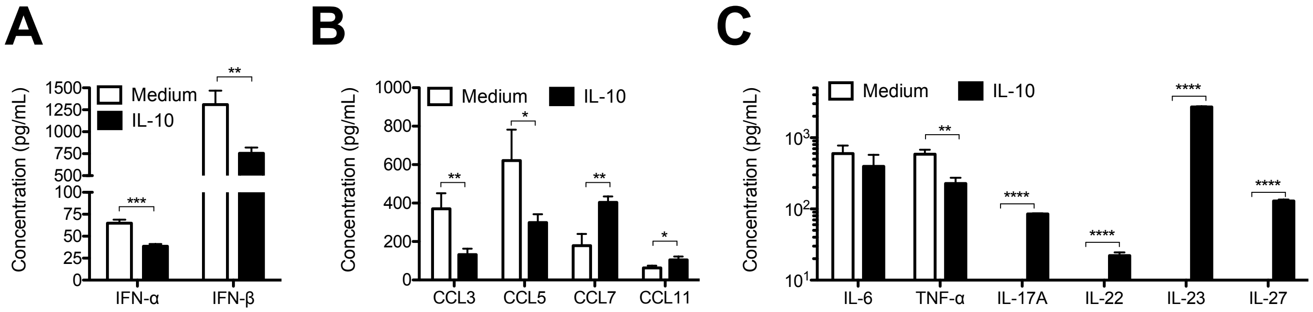

Figure 6 : IL-10 altered the response of neonatal AMs to *in vitro* RSV infection.

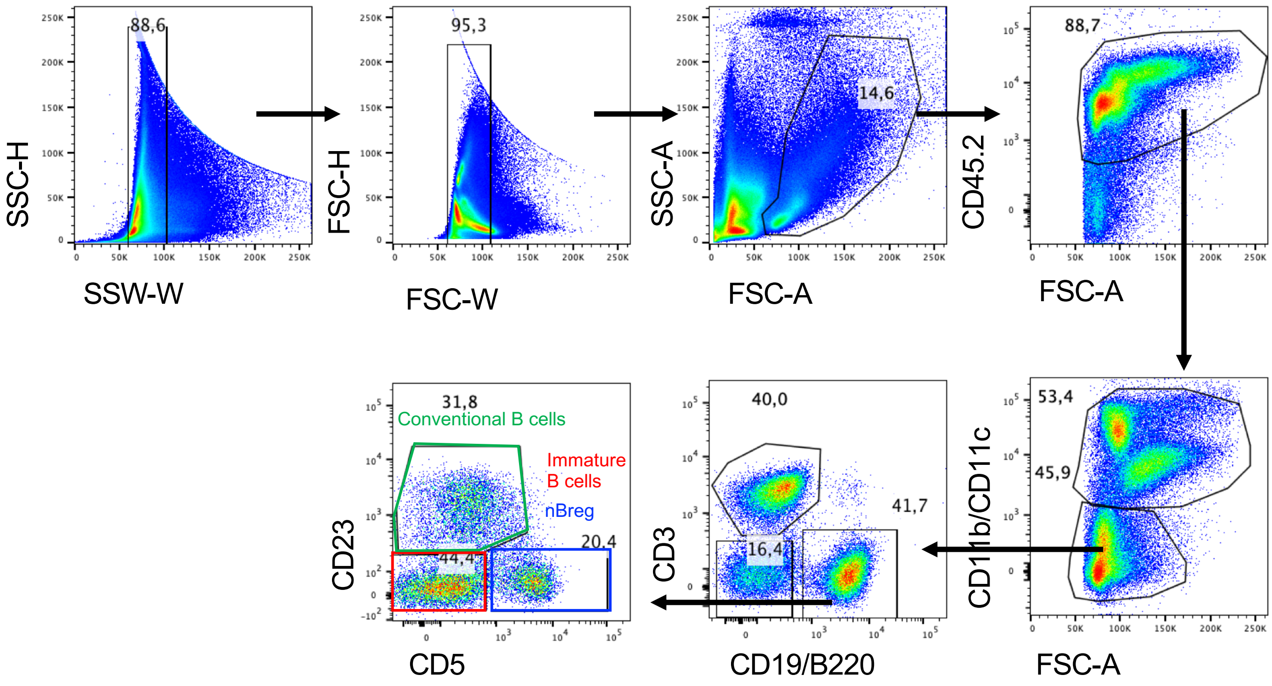

Supplemental Figure 1 : gating strategy to analysed for B cell populations by flow cytometry.

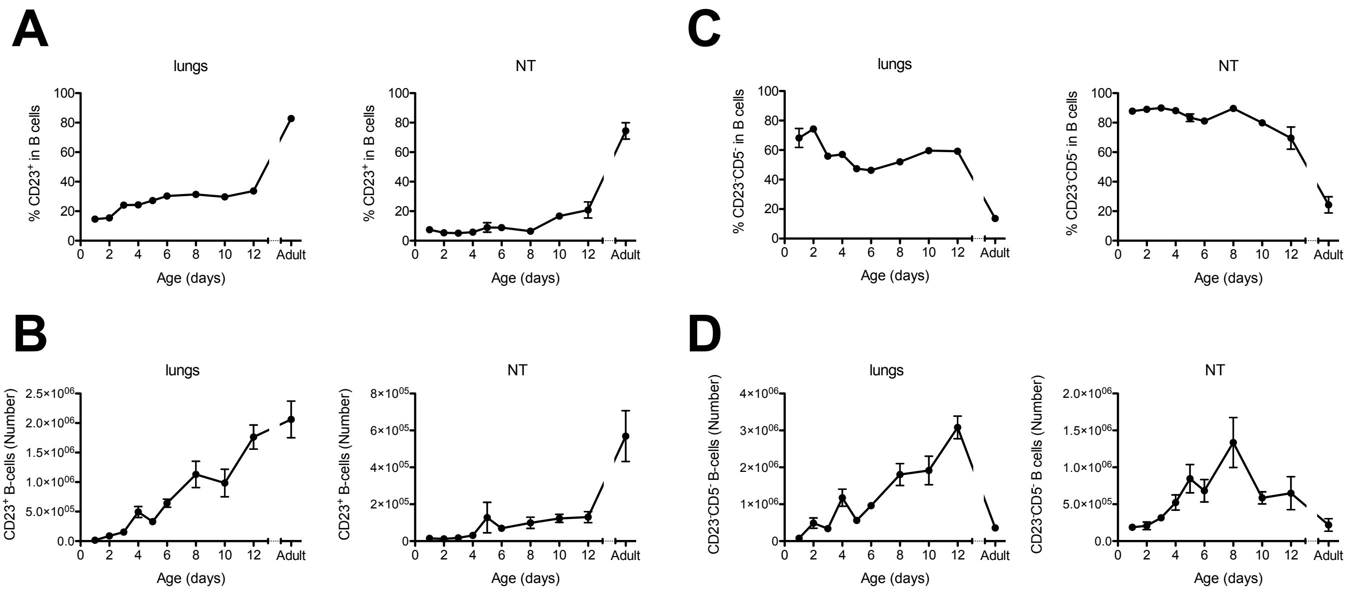

Supplemental Figure 2 : analysis of conventional and immature B cells subsets in the lungs and NT of neonates compared with adults

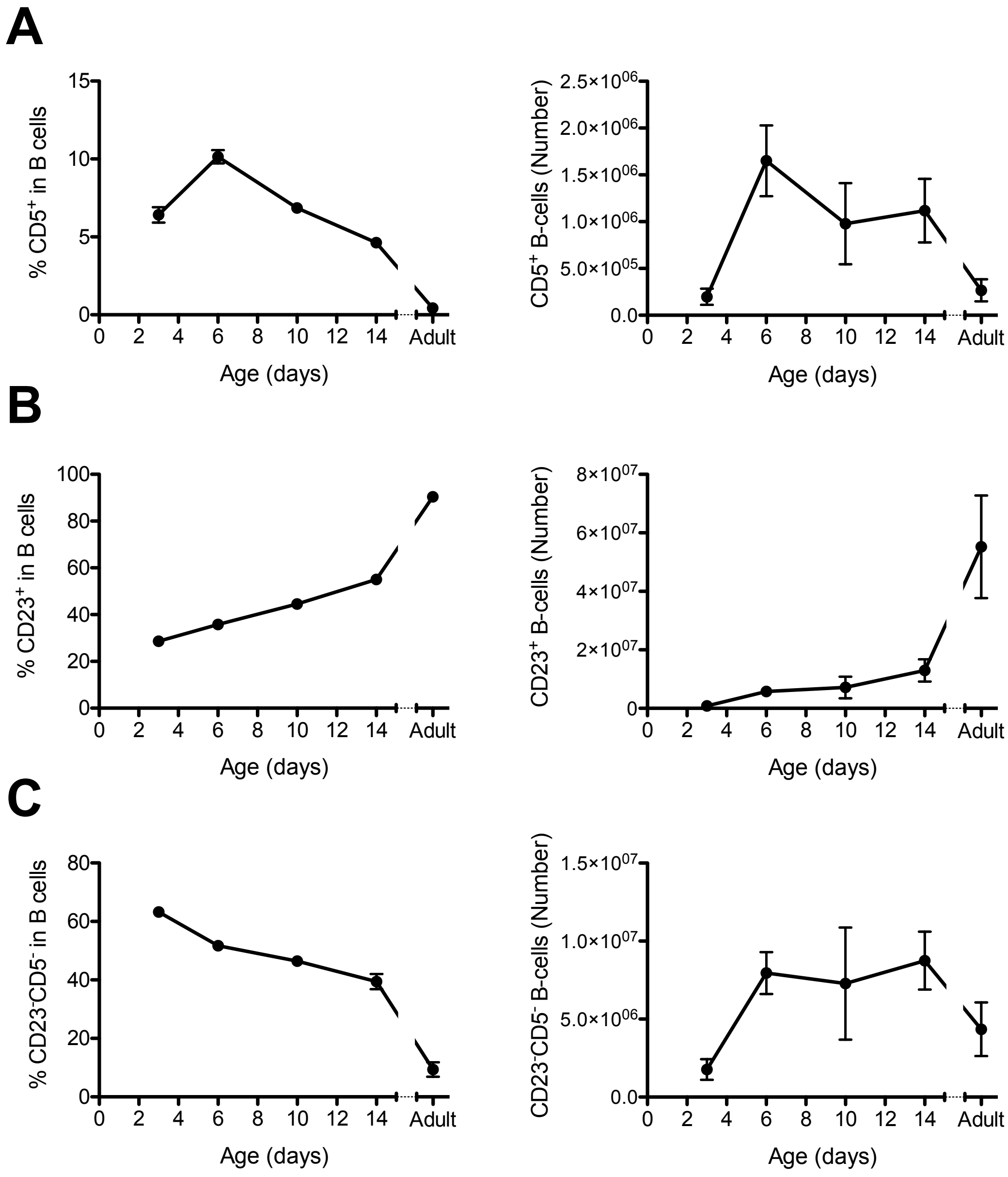

Supplemental Figure 3 : analysis of B cell subsets in the spleen of neonates compared with adults

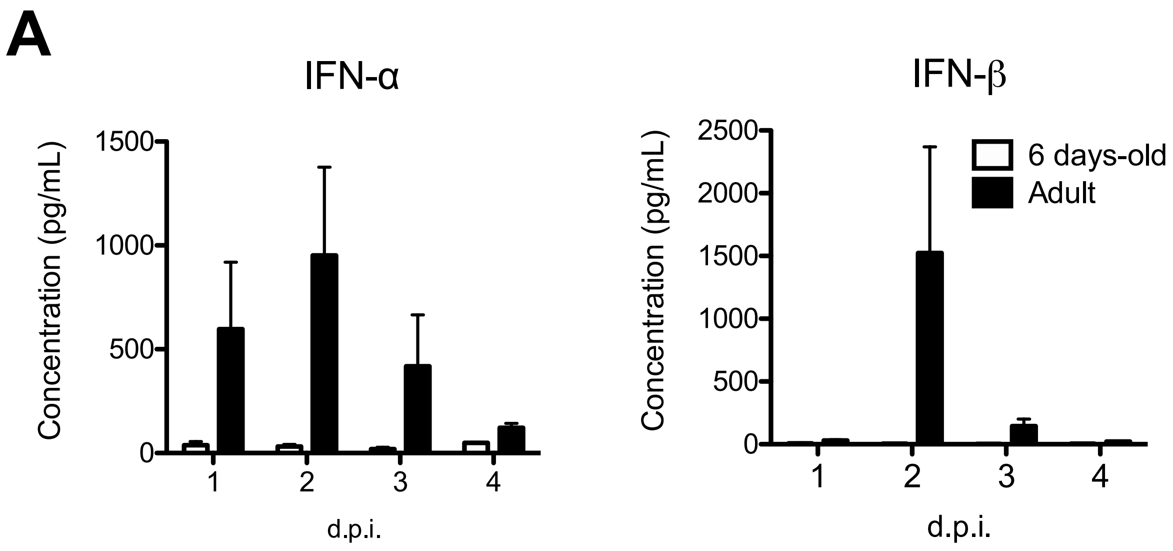

Supplemental Figure 4 : Differential IFN-I kinetic response to RSV infection between neonates and adult mice.

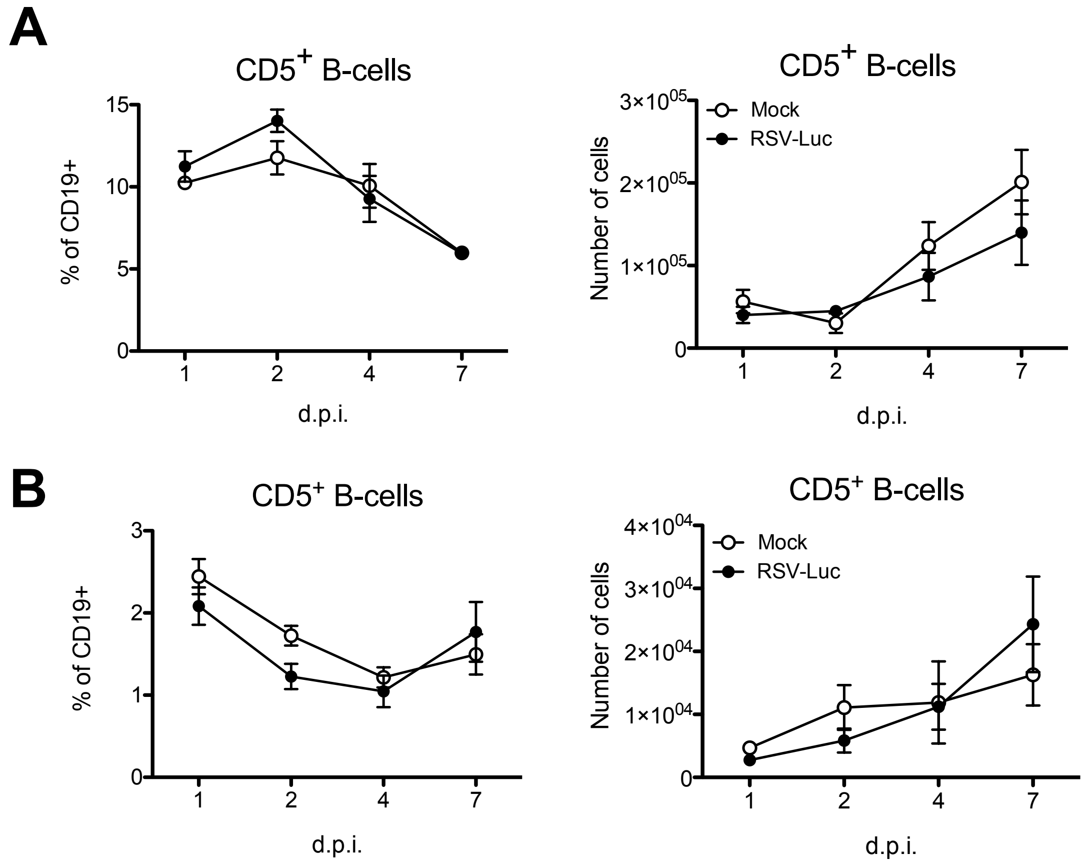

Supplemental Figure 5 : RSV infection in neonatal mice does not impact the presence of CD5+ B-cells in the respiratory tract.
